# Patterns of Multiple Risk Exposures for Low Receptive Vocabulary Growth 4-8 Years in the Longitudinal Study of Australian Children

**DOI:** 10.1371/journal.pone.0168804

**Published:** 2017-01-23

**Authors:** Daniel Christensen, Catherine L. Taylor, Stephen R. Zubrick

**Affiliations:** 1 Telethon Kids Institute, Subiaco, Western Australia, Australia; 2 Graduate School of Education, University of Western Australia, Nedlands, Western Australia, Australia; University College London, UNITED KINGDOM

## Abstract

Risk exposures and predictions of child development outcomes typically estimate the independent effects of individual exposures. As a rule though, children are not exposed piecemeal to individual or single risks but, rather, they are exposed to clusters of risk. Many of these clusters of risks are better thought of as comprising a developmental “circumstance” with a substantial duration, over which period, additional risk exposures also accumulate. In this paper we examined the distribution of 16 single risk exposures for low language ability using latent class analysis across a sample of approximately 4000 children from the Longitudinal Study of Australian Children. The best fitting model identified six distinct classes. 46% of children were in a Developmentally Enabled group, 20% were in a group typified as Working Poor families, 10% of children were in group typified as Overwhelmed group, 9% of children were in a group defined by Child Developmental Delay, 8% of children were in a group defined by Low Human Capital, and 7% of children were in a group defined by Resource Poor non-English Speaking background families. These groups had quantitatively and qualitatively distinct patterns of risk factors and showed different onward trajectories of receptive vocabulary. Our results demonstrate a range of multiple risk profiles in a population-representative sample of Australian children and highlight the mix of risk factors faced by children. Children with distinct patterns of risk factors have different onward trajectories of receptive vocabulary development.

## Introduction

Language, its acquisition and onward development, comprises a foundational human ability that, once established, grows and is used for the remainder of life [1]. It is a central milestone in early infant and child development and it features without fail as an area of focus across the range of public health, child care and early childhood education initiatives aimed at preventing developmental disadvantage and promoting optimal attainment of capabilities in children [2]. It is understandable that those who seek to improve lifecourse outcomes from an early age would wish to predict the influences on language acquisition and its onward development to design effective targeted and universal child development interventions.

This ambition is not without significant challenge. Epidemiological studies reveal remarkable variation between children in the emergence and growth of their language as measured by receptive or expressive vocabulary. Prediction of late language emergence at age 2 from information gathered at points earlier in pregnancy, birth and development is very poor [3]. Moreover, late language emergence does not portend onward delayed development for the vast majority of these children. Many of these children simply catch-up with their peers between the ages of 2 and 4[4] while some who were not late talkers go on to experience later language difficulties[5].

It might be thought that the large observable variability in language development of infants and very young children narrows as they emerge from infancy and enter preschool and primary school, thus improving predictability. Christensen et al. [6] investigated the extent to which low receptive vocabulary ability at 4 years was associated with onward low receptive vocabulary ability at 8 years, and estimated the predictive utility of a multivariate model that included relevant child, maternal and family risk factors measured at 4 years. They concluded that the positive and negative predictive values arising from these model was too low to be of practical use in selecting children for targeted intervention opportunities.

For professionals in public health and more broadly across paediatrics, allied health, child care and education, the desire to design and deliver effective interventions, to intervene early, and to reach the children most in need, must then be balanced against these converging findings across numerous large-scale longitudinal studies of infants and young children that predictive utility of onward outcome is poor [7]. These studies give caution for approaches that narrowly target children with these risks as opposed to interventions that are proportionately or progressively universal [8,9]. Risk exposures and predictions of outcomes arising from multivariate models have a natural emphasis on estimating the independent effects of individual exposures. As a rule though, children are not exposed piecemeal to single risks but, rather, they are exposed to clusters of risks. Many of these clusters of risks are better thought of as comprising a developmental “circumstance” with a substantial duration, over which period, additional risk exposures also accumulate.

In this paper we ask, are there clusters or classes of risk exposures that, when defined, would give rise to better universal or progressive universal targeting of language development interventions for young children? If so, how are these best characterized?

## Methods

The Longitudinal Study of Australian Children (LSAC) is a national longitudinal study that commenced in 2004. The study design is enumerated in detail elsewhere[10–13].

The study uses a cross-sequential design of biennial face-to-face visits with the family and study child. In this study we used data from the child cohort collected across three waves. The ages of the children at each wave are described in Table 1. For simplicity we refer to the children at the three waves as aged four, six, and eight years. The child cohort comprised 4983 children at four years, 4464 children at six years, and 4331 children at eight years.

**Table 1:** Sample size at each wave, children’s ages, and PPVT scores with available sample.

	Sample at each wave			Measures			
Wave	N	Child’s age in months			N^1^	Mean (SD)	Range
		Median	Range	Measure			
1	4983	57	51–67	PPVT	4406	65 (6.0)	28–85
2	4464	82	75–94	PPVT	4317	74 (5.0)	46–92
3	4331	105	95–119	PPVT	4273	78 (5.0)	45–106

The initial study sample was designed to be representative of Australian children within the selected age cohort, proportional to the regional distribution of children in the Australian population[11,12]. Analyses show that the initial sample was broadly representative when compared with 2001 Australian Census data, but slightly under-representative of families who were single-parent, non-English speaking and living in rental properties. Attrition somewhat increased these biases[13,14].

The risk framework uses all 4983 children who participated in the study at age four, while the analytic sample for the longitudinal analyses varies based on item level missingness.

### Ethics

The study has ethics approval from the Australian Institute of Family Studies Ethics Committee. As the study children were all minors at the time these data were collected, written informed consent was obtained from the caregiver on behalf of each of the study children. The signed consent forms are retained by the field agency (the Australian Bureau of Statistics).

### Measures

#### Outcomes

The outcome of interest in this paper was the child’s receptive vocabulary at ages four, six and eight. Receptive vocabulary was measured by the Adapted Peabody Picture Vocabulary Test-III (PPVT) score, which provides a continuous measure of language ability. The Adapted Peabody Picture Vocabulary Test-III (PPVT) is a test of receptive vocabulary designed for the LSAC study [15]. The Adapted PPVT-III is a shortened version of the PPVT—III [16]. The Adapted PPVT-III was administered directly to each child during the home interview. For each word presented, the child was shown a card containing four pictures and was asked to point to the picture corresponding to the word (e.g., “Show me wrapping”).

Table 1 contains the mean scores with SDs and associated ranges for the Rasch scaled PPVT.

#### Measures of risk

This paper uses 16 single risk factors. These risk factors were established as having a substantive association with intercept or slope of receptive vocabulary from ages four to eight in a growth curve model, based on a Cohen’s d of 0.30.[14]

The risk factors comprised five child factors (Study Child Indigenous status, low birthweight, low school readiness, low child persistence, and high child reactivity), five maternal factors (teenage mother at birth of study child, maternal psychological distress, low maternal education, maternal unemployment, and low parenting consistency), and six family factors (Mother non-English speaking background, four or more siblings, lowest family income, healthcare card, neighbourhood disadvantage, and no reading to the study child in previous week). These factors have been described in detail elsewhere [14]. For the purposes of this analysis, we have dichotomised each variable into an ‘at risk’ group and a reference, lower risk group. The development of these risk factors is described in S1 Appendix, and the risk factors are summarised in Table 2.

**Table 2:** Descriptive statistics for risk factors.

Risk Factor	% at risk in sample	n at risk in sample	Average additional risks
**Study Child**			
Study Child Indigenous status^a^	3.8	187	4.0
Low birthweight	6.5	317	2.9
Child in lowest quintile of school readiness	15.2	760	3.0
Child temperament in least persistent quintile	11.8	589	3.2
Child temperament in most reactive quintile	12.4	618	3.0
**Mother**			
Teenage mother at birth of study child	2.9	144	4.0
Mother K6 symptomatic	16.2	665	3.2
Mother year 11 education or less	39.2	1937	2.7
Mother unemployed	43.3	2159	2.6
Low parenting consistency	14.8	728	3.4
**Family**			
Mother non-English speaking	15.7	778	2.7
Four or more siblings	4.1	206	3.8
Family in lowest quintile for income	17.5	818	3.7
Healthcare card	22.0	1095	3.5
Most disadvantaged quintile for neighbourhood disadvantage	21.9	1091	2.9
Child not read to all in last week	3.6	182	4.2

^a^ Study child is of Aboriginal or Torres Strait Islander descent

The total number of risks was defined as the sum of these risks, giving each child a potential number of risks from 0 to 16 (Table 3). The accumulation of risk factors across the population is discussed in more detail in S2 Appendix.

**Table 3:** Cumulative distribution of multiple risks across study sample, wave 1.

Risks	Frequency	Percent	Cumulative Frequency	Cumulative Percent
0	719	14.4	719	14.4
1	1205	24.2	1924	38.6
2	994	20.0	2918	58.6
3	725	14.6	3643	73.1
4	554	11.1	4197	84.2
5	339	6.8	4536	91.0
6	245	4.9	4781	96.0
7	125	2.5	4906	98.5
8	51	1.0	4957	99.5
9	20	0.4	4977	99.9
10	4	0.1	4981	100.0
11	2	0.04	4983	100

### Statistical analysis

The analysis proceeded in three steps: 1) a latent class analysis (LCA) was used to identify and describe profiles of risk for receptive vocabulary children in the LSAC, using risk factors measured when the child was four years old; 2) information from the latent class model was used to assign children to risk profiles; and 3) receptive vocabulary outcomes were assigned children based on their risk profiles.

LCA was applied to the dichotomised measures of risk [17,18]. The technique aims to identify substantively meaningful classes within which participants have a similar response pattern on a set of observed variables [19]. LCA estimates class probability parameters, which estimate the proportions of the population that fall in each class, and the item response probabilities, which provide information on the probability of an individual in that class to endorse the observed measures [18]. All LCA analyses were conducted using SAS PROC LCA Version 1.2.4 [20,21].

Model fit in latent class analysis is guided by statistical measures of model fit and by the subjective interpretation of the assigned classes [22].

The AIC (Akaike information criterion) and BIC (Bayesian information criterion) were used as the statistical measures of model fit in this paper. Lanza et al. [23] advise that each class should be distinguishable from the others on the basis of the item-response probabilities, no class should be trivial in size, and it should be possible to assign a meaningful label to each class.

Information from the LCA model was used to assign children to risk profiles. Individuals were assigned to latent classes based on their maximum posterior probability [22].

Using each child’s assigned risk profile, the child’s PPVT score at ages four, six and eight was assessed by a growth model, conducted in PROC MIXED in SAS version 9.4 [24]. Our growth curve modelling utilises a two-level nested structure. Level one is the within-person model while level two is the between-person model. This approach has been described extensively elsewhere [14,25]. These models estimate intercept and slope effects of receptive vocabulary development in relation to the derived risk profiles.

## Results

Table 2 shows the percentage and number of children with each single risk exposure in the sample at age four and the average additional risk exposures associated with each single risk exposure. For example, children in the lowest quintile of school readiness were exposed to three additional risk factors.

Sixteen possible risk factors allows for 2^16^ (65,536) possible combinations of risk factors. Of these, 1585 were observed in the data. Table 3 shows the distribution of risk exposures across the population. The average number of risk exposures per child was 2.5. 14% of children were exposed to none of the designated risk factors, almost two thirds of children had two or more risks exposures, with 42% of children experiencing three or more risks.

AIC and BIC were examined to determine the ideal number of classes in the model (see S3 Appendix). Given that BIC suggested a 6-classes model, and AIC suggested an 11-class model, all options between 6 and 11-classes were considered. A 6-class model was selected on the basis of statistical criteria and the fact that this model produced a clear distinction between classes.

Table 4 shows the probability of having a single risk exposure by class membership for each of the latent classes. We also provided the estimated population probability average for each of the risks. This allows comparisons to be made between the reference group (Developmentally Enabled) and each of the remaining groups but it also allows comparison with the population average probabilities. Because of the binary nature of the predictor, these probabilities can be interpreted as proportions.

**Table 4:** Conditional probabilities and distributions of risks, 6-class latent class analysis.

Class	Developmentally enabled (ref.)	Working poor	Overwhelmed	Developmental delay	Low human capital	Resource poor non-English speaking	Population average
Proportion	**0.46**	0.20	0.10	0.09	0.08	0.07	
SE	**-0.04**	-0.03	-0.01	-0.02	-0.01	-0.01	
	Item response probabilities						
Child Indigenous status	0.01	0.05	0.19	0.01	0.03	0.01	0.04
Low Birthweight	0.05	0.06	0.12	0.09	0.08	0.07	0.06
Child low school readiness	0.06	0.21	0.31	0.37	0.14	0.11	0.15
Child low persistence	0.04	0.07	0.24	0.47	0.07	0.16	0.12
Child high reactivity	0.09	0.03	0.30	0.40	0.05	0.12	0.12
Teenage mother	0.00	0.04	0.12	0.01	0.09	0.00	0.03
Maternal mental health distress	0.10	0.08	0.44	0.26	0.13	0.42	0.16
Low mother education	0.18	0.60	0.84	0.42	0.53	0.37	0.39
Mother not employed	0.26	0.44	0.83	0.38	0.65	0.75	0.43
Low parenting consistency	0.06	0.09	0.44	0.28	0.11	0.35	0.15
Non-English background	0.13	0.00	0.13	0.08	0.12	0.98	0.16
> = 4 Siblings	0.00	0.09	0.14	0.01	0.00	0.11	0.04
Lowest income	0.02	0.00	0.59	0.05	0.97	0.33	0.18
Healthcare card	0.04	0.19	0.61	0.10	0.84	0.35	0.22
Area disadvantage	0.11	0.32	0.41	0.19	0.26	0.35	0.22
Low book reading	0.00	0.03	0.14	0.03	0.02	0.14	0.04
**Mean risks**	1.05	2.84	6.09	3.78	3.86	4.70	2.46

The six classes differed in prevalence from 46% in class one to 7% in class six.

The first group (i.e. reference group) was typified as Developmentally Enabled. This group made up 46% of the sample. On average, each child in this group was exposed to 1.0 risk at age four. Its distinguishing feature was a consistently lower than population-average proportion for each of the risk factors, with a likelihood of zero for teenage motherhood, four or more siblings, and the study child not being read to at all.

The second group (20%) comprised Working Poor families. On average, each child in this group was exposed to 2.8 risks. Relative to the population proportion, this group had a similar proportion of unemployed mothers (44%) when the child was 4 years old. Children in this group were more likely to exhibit low school readiness, have mothers with low education, have four or more siblings, and live in disadvantaged areas. Non-English speaking status families were not in this group.

The third group (10%) was typified by multiple risk factors across all domains, which we termed Overwhelmed. On average, each child in this group was exposed to 6.1 risks. Relative to both the Developmentally Enabled and the population average, this group had an increased likelihood of all risk factors, other than maternal non-English speaking background.

The fourth group (9%) was characterised by a combination of child developmental factors, which we termed Developmental Delay. On average, each child in this group was exposed to 3.8 risks. Higher proportions of children in this group had low temperamental persistence (47%) and reactive temperament (40%).

Making up 8% of the sample, the fifth group was typified by Low Human Capital and can be contrasted to those in the Developmentally Delayed circumstance by a higher proportion of teenage mothers (9%) and maternal low education (53%). Maternal unemployment (65%) is very high relative to the population proportion. On average, each child in this group was exposed to 3.8 risks. This group has the highest proportion (97%) of families in the lowest income quintile and has the highest healthcare card use (84%). Importantly, the proportion of children in the Low Human Capital group with low school readiness (13%) is comparable to the population average.

The sixth group (7%) we describe as Resource Poor non-English Speaking. On average, each child in this group was exposed to 4.7 risks. This group included 44% of the non-English speaking mothers. It had an increased proportions of mothers with maternal psychological distress (42%), low parenting consistency (35%), four or more siblings (11%), low income (33%), healthcare card (35%), neighbourhood disadvantage (35%), and not reading to the study child (14%). This group did not show any increased likelihood for study child Indigenous status, child reactive temperament, or teenage motherhood.

Table 5 shows the results of fitting a growth model for children in each of the six classes. The youngest child in the study was 50 months old at wave one, so this age was set as the baseline or intercept. The median ages at waves one, two and three were 57, 82 and 105 months, so we show the average predicted PPVT for each group at the mid-point of each wave. The final columns in Table 5 show the difference between each group and the reference class at 105 months of age. As differences in PPVT points are not immediately intuitive, differences between groups have been converted to a difference in months, which is based on the growth rate of children in the reference group.

**Table 5:** Growth model on Latent Classes (LCA), peabody picture vocabulary scores from ages 4 to 8^a^.

				PPVT scores at 50, 57, 82 and 105 months				Difference at105 months	
LCA Group	Intercept	Months	Slope	50	57	82	105	Points	Months
Developmentally enabled (ref.)	64.4*** (64.2, 64.6)		0.2808*** (0.2759, 0.2857)	64.4 (64.2, 64.6)	66.4 (66.1, 66.6)	73.4 (73.2, 73.5)	79.8 (79.7, 80.0)		
Working poor	-1.6*** (-2.1, -1.1)	-5.8	-0.0009^n.s.^ (-0.0110, 0.0092)	62.8 (62.4, 63.2)	64.7 (64.4, 65.1)	71.7 (71.4, 72.0)	78.2 (77.8, 78.5)	-1.7*** (-2.0, -1.3)	-5.9
Overwhelmed	-5.3*** (-6.0, -4.6)	-18.9	0.0293*** (0.0149, 0.0438)	59.1 (58.5, 59.7)	61.3 (60.7, 61.8)	69.0 (68.6, 69.4)	76.1 (75.6, 76.7)	-3.7*** (-4.2, -3.1)	-13.1
Developmental delay	-2.7*** (-3.4, -1.9)	-9.6	0.0120 ^n.s.^ (-0.0035, 0.0277)	61.7 (61.0, 62.4)	63.8 (63.1, 64.4)	71.1 (70.6, 71.6)	77.8 (77.3, 78.4)	-2.0*** (-2.6, -1.4)	-7.2
Low human capital	-1.7*** (-2.4, -1.1)	-6.1	0.00736 ^n.s.^ (-0.0064, 0.0211)	62.7 (62.1, 63.3)	64.7 (64.2, 65.2)	71.9 (71.5, 72.3)	78.5 (78.0, 79.0)	-1.3*** (-1.8, -0.8)	-4.7
Resource poor NESB	-7.4*** (-8.1, -6.7)	-26.3	0.0813*** (0.0656, 0.0969)	57.0 (56.3, 57.7)	59.5 (58.9, 60.2)	68.6 (68.1, 69.0)	76.9 (76.4, 77.5)	-2.9*** (-3.5, -2.3)	-10.4

^a^ figures in parentheses are 95% confidence intervals;

*** p < 0.001;

^n.s.^ non-significant

For the second to sixth groups the intercept effect shows how far behind these groups are when the child is aged 50 months. All of their intercepts are negative relative to the developmentally enabled—they are all lagging behind in their vocabulary. However, at 50 months there are striking differences in the magnitude of this delay: The resource poor non-English speaking group is over two years behind the developmentally enabled group, followed by the children in the overwhelmed group who are about 20 months behind. Children in the working poor and in the low human capital groups are about half a year behind those who are developmentally enabled.

Six years later the children in all of these groups also demonstrate delayed vocabulary growth relative to the developmentally enabled. However, the growth over six years has been differential. Starting 26 months behind their developmentally enabled counterparts, the resource poor non-English speaking children have more than halved their disadvantage to a delay of 10 months, demonstrating the largest rate of growth in their vocabulary after the developmentally enabled. The children in the Overwhelmed group are now the most delayed (-13 months), although they have reduced this delay by almost half a year relative to where they started. Children in the working poor group demonstrated no differential growth finishing as they started—half a year behind the developmentally enabled. Some slight gains were made in vocabulary growth for the groups with developmental delay and low human capital.

## Discussion

This study considers whether there are clusters or classes of risk circumstances that, when defined, would give rise to better targeting of language development interventions for young children. Our findings show that developmental risk circumstances can differ substantively from one another. Sixteen risk factors representing a mix of child, maternal and family factors were modelled via latent class analysis and six distinct groups of risk circumstances were identified. The average number and type of risks experienced by children in these groups differed considerably and no group was defined exclusively by the presence or absence of a single risk factor.

How might these circumstances guide considerations and policies for designing universal and universally proportionate developmental interventions?

Nearly one-half (46%) of the children could be characterised as developmentally enabled. While this group is not without exposure to some of the modelled risks, they are identifiable on the basis of their very low exposure to risk. These children are in families which can be described as having a reserve capacity or “generalised psychosocial capacities” [26,27] that can be used to buffer the child against risk, directly ameliorate the effects of exposure or prevent the exposure all-together. It could be expected that these families know how to use child development opportunities when they need them and are proactive in creating these circumstances for their children. In population terms this is a group that policy makers and service providers would like to “grow”.

The next most common developmental risk circumstance is that of the working poor family making up 20% of the sample. They are on average exposed to 2.8 risks when first seen at age 4. These children are in families that face higher risks and they do so with a lower level of reserve capacity. Over the four year period these children consistently remained six months behind their developmentally enabled peers. Owing to their work commitments, parents of these children are likely to have less time for the care of their children; they are less likely to be eligible for, or receive a lower level of, income support; and they have less disposable income to divert to child care and developmental resources and opportunities. In policy terms, there is broad consensus that income inequality is negatively associated with child achievement [28]. Moreover, where policies seek to replace income transfers with work programs these programs need to alleviate income poverty in order to reduce harsh, controlling parenting and consequent lower cognitive performances in children [29].

The remaining four groups comprise about 35% of the rest of the sample and are distributed across quite different risk circumstances.

Consistent with previous work in Australia and overseas, we find that there is a group of children who are exposed to multiple risks. These families (10%) are in the Overwhelmed group. Families of these children commonly and prominently feature in the policy interests of governments. Low maternal education, unemployment, lowest income and particularly, area disadvantage, set the context for very poor child development in this group with high proportions of children receiving inconsistent parenting and lower levels of parental reading. While these families are relatively easy to identify and appear across multiple human service portfolios their capacity for engagement in child development opportunities is limited. A major policy challenge then is configuring child development opportunities to occur where these families are more likely to live or appear rather than simply assume the problem lies with their willingness to engage.

Sharing the equal lowest position in terms of child development are the 9% of families in which, the principal characteristic is the developmental delay of the child. Children in these circumstances are likely to appear across a range of medical, other health services, early child care and education settings and may not be identified until formal enrolment in education.

About 8% of children lived in circumstances we characterised as Low Human Capital. The presence of higher proportions of young mothers in this group invites interventions that change both parental capacities as well as those of the children themselves, and enable this across different settings.

Finally, the risk circumstances of 7% of the remaining children are notable for the presence of maternal non-English speaking status in the presence of very high maternal mental health distress, inconsistent parenting style, low hours of employment, larger family size, low income, neighbourhood disadvantage and infrequent parental reading. On average, children in these families were exposed to 4.7 risks at age 4. It is important to note that although this group is typified by the high likelihood (p = 0.98) of maternal non-English status, not all non-English speaking mothers were in this group, with 56% of non-English speaking mothers belonging to one of the other classes including the Developmentally Enabled. Encouragingly, the children in this group had rapidly growing vocabulary performance. A longer period of observation may have seen this gap further close.

We asked at the outset whether knowledge about these classes of risk might provide guidance in positioning universal or progressively universal opportunities and expectations for children in need of these. In our view, human service settings that offer progressively universal interventions still require some level of identification of children for such services as these intensify to meet need. What level of delay, need, or circumstance would qualify the child and family for this? Our findings can only provide early insights into how this might be done in the absence of corroborating confirmation and comparisons with other studies.

First, while the focus of our analysis was on the period of development from 4 years to 8 years, children in all other groups than the Developmentally Enabled started with lower levels of development. Identifying and intervening with the families that have multiple risks across a broad front of human service need offers an early point for progressing intervention intensification for these families prior to age 4.

Second, in population terms, children in the Working Poor group were not able to close a six month gap in development over four years. They represent the single largest group (20%) after the reference group. In considering interventions this points to changing policies and programs that govern threshold barriers to access of support and services and or that enable direct or indirect redress of inequities in family income, parental education, early childhood education and area disadvantage.

Third, identification of children who present with persistent developmental delay in the absence of other family or parental economic and psychosocial risks would best be achieved through repeated monitoring, initiation of developmental opportunities for enrichment, and appropriate developmental support.

Fourth, with regard to children of non-English speaking families, many of these children go on to close the developmental gap and others, very notably in resource poor families, struggle to catch up with their English speaking peers. None-the-less, even the children in this latter group make substantial gains relative to any other group. We have previously noted the that non-English speaking children catch up completely to their English-speaking counterparts in a multivariate growth curve model[14]. Our findings here though illustrate the importance of considering co-morbid or overlapping patterns of risk in these children and their families [30].

Several limitations of this work should be noted. Although influenced by substantive interpretation, LCA is a data-driven technique. This can make findings sample dependent, and can makes comparisons between studies more complicated. We found it challenging to typify the classes in a simple fashion. While the title-descriptions of these circumstances are a convenient short-hand, the tables reveal an overlap in the risk circumstances that belies this convenience. This, combined with the subjective assessment of group-meaning means that the assigned classes should not be reified[17].

Finally, the policy implications of our findings require further exploration. Our previous work has shown that a well-fitted multivariate model does not necessarily predict child language outcomes across time with even reasonable predictive utility[6,31]. The approach we present here moves away from a concern with individual prediction, instead, conceptualising human circumstances that might be considered in the development of policies effecting whole populations. Relative to the Developmentally Enabled group, all of the other groups would benefit from micro-economic policies that enable a more equitable distribution of opportunities for parental education and training and employment. Improving developmental resources and opportunities in disadvantaged areas is also supported by these data. Low school readiness, on the other hand, offers opportunities for more targeted approaches among the circumstances of the Working Poor, Overwhelmed, and Developmentally Delayed.

The findings here suggest no quick fixes or silver bullets. They do however invite more considered population approaches, and broader vigour in implementing public policies and strategies to address deepening inequalities in the financial and material circumstances of families and their children. These approaches will need to operate over extended time frames with more coordinated and navigable pathways that intersect with family and child development opportunities.

## Supporting Information

S1 Appendix Development of risk factors. (DOCX)

S2 Appendix Distribution of risks. (DOCX)

S3 Appendix Model fit. (DOCX)
