# Correction: Patterns of Multiple Risk Exposures for Low Receptive Vocabulary Growth 4-8 Years in the Longitudinal Study of Australian Children

**DOI:** 10.1371/journal.pone.0172377

**Published:** 2017-02-13

**Authors:** The PLOS ONE Staff

The Data Availability statement for this paper is incorrect. The correct statement is: This paper uses unit record data from Growing Up in Australia, the Longitudinal Study of Australian Children. The study is conducted in partnership between the Department of Social Services (DSS), the Australian Institute of Family Studies (AIFS) and the Australian Bureau of Statistics (ABS). The findings and views reported in this paper are those of the author and should not be attributed to DSS, AIFS or the ABS.

Data are available from the Longitudinal Study of Australian Children authors. They are contactable via: http://www.growingupinaustralia.gov.au/data/dataaccessmenu.html

Users may apply for individual or organisational licenses. There is a fee for the licenses and release of data. The data analysed in this project were accessed via an organisational license. SRZ is the Data Manager for the Telethons Kids Institute and is responsible for maintaining the organisational deed. DC and CLT signed a Deed of Confidentiality to access these data, which SRZ returned for authorisation to the Department of Social Services. No data were analysed (other than derived analytic variables) which are not available to licensed users of the data.

There are errors in the Funding section. The correct funding information is as follows: This work was supported by a grant from the Australian Research Council (CE140100027). The publisher apologizes for the errors.
