# Supplementary material for: Patterns of Multiple Risk Exposures for Low Receptive Vocabulary Growth 4-8 Years in the Longitudinal Study of Australian Children
Source: PLoS One. 2017 Jan 23;12(1):e0168804. doi: 10.1371/journal.pone.0168804 (PMC5256896; doi:10.1371/journal.pone.0168804)
Supplement: S1 Appendix — (DOCX) [file pone.0168804.s001.docx]

# S1 Appendix: Development of risk factors

Taylor et al. [1] identified 16 single risk exposures that had a substantive association with receptive vocabulary from ages four to eight in a growth curve model. For the purposes of this analysis, a substantive association was defined as an effect on intercept or slope equal to or greater than a Cohen’s d of = 0.30[1].

The risk factors comprised five child factors (Study Child Aboriginal or Torres Strait Islander status, low birthweight, school readiness, child persistence, and child reactivity), five maternal factors (teenage mother at birth of study child, maternal psychological distress, maternal education, maternal work hours, and maternal parenting consistency), and six family factors (Mother non-English speaking background, four or more siblings, family income, healthcare card, neighbourhood disadvantage, and reading to the study child).

All risk factors are measured when the study child as aged four. Consistent with our previous work, where there are not *a priori* external referents for risk (such as low birthweight) we have considered children in the ‘worst performed’ quintile as at the greatest risk. This approach to parameterization is empirically substantiated by our results[1,2].

Study child minority race has previously been identified as an independent risk factors for low receptive language ability. A small proportion of children (n= 187; 3.8%) were of Aboriginal and/or Torres Strait Islander decent and were coded to distinguish them from those who were not.

Primary carers were asked to report their child’s birth weight which was subsequently coded into those children who were born with low birth weight (<2500 grams; 6.5%) and those who weighed more than this (>= 2500 grams).

Each study child’s school readiness was directly assessed at four years using the *Who Am I? (WAI)* [3]. The WAI comprises 11 items in which children write their names, copy shapes and write words and numbers. It has been calibrated for use in the LSAC and has high internal reliability (0.89), and excellent distributional properties[3]. In this study, children in the lowest quintile of WAI performance are considered at risk (n = 760; 15.2%).

Child persistence and reactive temperament was measured at four years with the Short Temperament Scale for Children (STSC) [4]. The STSC was developed for us in the LSAC. Each temperament dimension was assessed through parent report using four items, rating the frequency of the behaviours on a 6-point Likert scale of occurrence from ‘‘almost never’’ to ‘‘almost always’’. In this report, children in the most reactive quintile (n = 618; 12.4%) and the least persistent quintile (n = 589; 11.8%) are considered at risk.

The children of mothers who were teenage at the birth of the study child (n = 144; 2.9%) were considered at risk.

In this study, we used the Kessler-6 (K6) scale to measure maternal non-specific psychological distress. Women with scores of eight or more were classified as having symptomatic psychological distress. This threshold is consistent with other studies [5-7] using the K6.

In Australia, at the time of this study, 10 years of education was compulsorily mandated. Mothers with year 11 or less were considered at risk (n = 1937, 39.2%).

We used total hours of paid maternal employment to distinguish mothers who were not in paid employment (zero hours), from those in part time and full time paid employment. 43.3% (n = 2159) of mothers were not in employment when the study child was aged four.

The parenting consistency of the mother was measured in a self-complete form developed for the LSAC [8,9]. Responses to each item were on a 5-point Likert scale, ranging from ‘‘almost never’’ to ‘‘always/almost always’’. Items for each measure were summed to create a composite score with higher levels representing more positive parenting characteristics. Children of mothers in the lowest quintile of parenting consistency (n = 728; 14.8%) were considered at risk.

As the focus of this study is explicitly on English language development and because language development is known to vary where more than one language is spoken in the home, we used the mother’s non-English speaking background (NESB) as a general indicator for language other than English spoken in the household at four years.

Children with 4 or more siblings (n = 206, 4.1%) were considered at risk for language development.

Families were asked to report their total weekly family income from all sources. Responses were partitioned into relatively equal quintiles. Those families earning under $600 per week (n = 818, 17.5%) were considered at risk.

Access to a health care card was considered as an additional measure of financial hardship. In Australia where income falls below a defined threshold and/or certain hardship criteria are met families also qualify for a health care card.

An area measure of socioeconomic disadvantage was also estimated for each participating family. The family home was coded with Socio-Economic Indicators for Area (SEIFA) disadvantage, indexed in quintiles – with families in lowest quintile considered at the greatest levels of disadvantage[10].

Finally, study children not read to at all in the last week (n= 182, 3.6%) were considered at risk.

# References

1. Taylor CL, Christensen D, Lawrence D, Mitrou F, Zubrick SR (2013) Risk factors for children’s receptive vocabulary development from four to eight years in the Longitudinal Study of Australian Children. PLoS ONE 8.

2. Christensen D, Zubrick SR, Lawrence D, Mitrou F, Taylor C (2014) Risk Factors for Low Receptive Vocabulary Abilities in the Preschool and Early School Years in the Longitudinal Study of Australian Children. PLoS One 9.

3. de Lemos M, Doig B (1999) Who am I? Developmental Assessment Manual. Melbourne: Australian Council for Education Research.

4. Sanson A, Prior M, Oberklaid F, Garino E, Sewell J (1987) The structure of infant temperament: Factor analysis of the Revised Infant Temperament Questionnaire. Infant Behavior & Development 10: 97-104.

5. Strazdens L, Shipley M, Broom D (2007) What does family-friendly really mean? Wellbeing, time, and the quality of parents' jobs. Australian Bulletin of Labour 33: 202-225.

6. Strazdens L, Shipley M, Clements M, O'Brien L, Broom D (2010) Job quality and inequality: Parents' jobs and children's emotional and behavioral difficulties. Social Science and Medicine 70: 2052-2060.

7. Lawrence D, Mitrou F, Zubrick SR (2011) Non-specific psychological distress, smoking status and smoking cessation: United States National Health Interview Survey 2005. BMC Public Health 11: 256.

8. Zubrick SR, Smith GJ, Nicholson JM, Sanson AV, Jackiewicz TA, et al. (2008) Social Research Policy Paper No. 34: Parenting and families in Australia. Canberra: Australian Government Department of Families, Housing, Community Services and Indigenous Affairs.

9. Zubrick SR, Lucas N, Westrupp E, Nicholson J (2014) Parenting measures in the Longitudinal Study of Australian Children: Construct validity and measurement quality, Waves 1 to 4. In: Services DoS, editor. Canberra: Department of Social Services.

10. ABS (2003) Information paper - Census of Population and Housing, Socio-Economic Indexes for Areas, Australia 2001. In: Statistics ABo, editor. Canberra: Australian Bureau of Statistics.
