# Supplementary material for: Patterns of Multiple Risk Exposures for Low Receptive Vocabulary Growth 4-8 Years in the Longitudinal Study of Australian Children
Source: PLoS One. 2017 Jan 23;12(1):e0168804. doi: 10.1371/journal.pone.0168804 (PMC5256896; doi:10.1371/journal.pone.0168804)
Supplement: S2 Appendix — (DOCX) [file pone.0168804.s002.docx]

# S2 Appendix: Distribution of risks

Figure 1 describes the population distribution of risks at age four. The observed data describe the distribution of risks observed in the study sample. The independent observations describes the distribution of risks that would have been observed, had each risk factor been independent of each other. This distribution was estimated via a Monte-Carlo simulation (n = 100,000,000), in which each case had a random likelihood for each risk factor. The observed data show a distribution which could be described as less equitable – there are more children observed with zero or one risks than would be expected if risks were independent, and there are more children with five or more risks than would be expected if risks were independent.

**Figure 1. Population distribution of risks.**

Table 1 describes the inter-relationships of risks when the child is aged four, estimated by finding odds ratios of having one risk factor based on the presence of another risk factor. It is also apparent that some risk factors tend to co-occur in overlapping patterns. For example, study child Indigeneity was associated with a substantially increased likelihood of teenage motherhood, low maternal education, and large family size, but was associated with a decreased likelihood of a non-English speaking background mother. Non-English speaking background mother was associated with increased odds of elevated psychological distress, and not reading to the study child but was associated with a decreased likelihood of low school readiness, teenage motherhood, and low maternal education. Child reactive temperament was associated with low persistent temperament, elevated mother psychological distress, and low parenting consistency, but was not significantly associated with a range of other risk factors.

**Table 1. Relationships between risk factors - odds ratios.**

|  | **Risk predicted** | | | | | | | | | | | | | | | |
| --- | --- | --- | --- | --- | --- | --- | --- | --- | --- | --- | --- | --- | --- | --- | --- | --- |
| **Risk Factor** | Study Child ATSI | Low birthweight | Child in lowest quintile of school readiness | Child temperament in least persistent quintile | Child temperament in most reactive quintile | Teenage mother at birth of study child | Mother K6 symptomatic | Mother year 11 education or less | Mother zero hours | Maternal consistency in lowest quintile | Mother NESB | Four or more siblings | Family in lowest quintile for income | Healthcare card | Most disadvantaged quintile for neighbourhood disadvantage | Child not read to all in last week |
| Study Child ATSI |  | 1.498 | 1.801*** | 0.925 | 1.373 | 4.985*** | 2.061*** | 3.734*** | 2.313*** | 3.045*** | 0.387*** | 3.309*** | 3.045*** | 2.085*** | 2.988*** | 2.116* |
| Low birthweight | 1.498 |  | 1.663*** | 1.117 | 1.386* | 1.638 | 1.249 | 1.263* | 1.157 | 1.032 | 1.142 | 1.433 | 1.546** | 1.774*** | 1.233 | 1.843** |
| Child in lowest quintile of school readiness | 1.801*** | 1.663*** |  | 2.690*** | 1.521*** | 1.108 | 1.1 | 1.735*** | 1.562*** | 1.503*** | 0.671** | 1.651** | 1.338** | 1.497*** | 1.533*** | 2.040*** |
| Child temperament in least persistent quintile | 0.925 | 1.117 | 2.690*** |  | 2.799*** | 1.282 | 1.896*** | 1.612*** | 1.410*** | 2.006*** | 1.112 | 0.989 | 1.384** | 1.346** | 1.283* | 2.073*** |
| Child temperament in most reactive quintile | 1.373 | 1.386* | 1.521*** | 2.799*** |  | 1.095 | 3.016*** | 1.292** | 1.206* | 2.277*** | 1.041 | 1.252 | 1.352** | 1.308** | 0.916 | 1.285 |
| Teenage mother at birth of study child | 4.985*** | 1.638 | 1.108 | 1.282 | 1.095 |  | 2.101*** | 4.519*** | 2.539*** | 1.860*** | 0.292*** | DNC | 4.595*** | 3.138*** | 2.083*** | 1.198 |
| Mother K6 symptomatic | 2.061*** | 1.249 | 1.1 | 1.896*** | 3.016*** | 2.101*** |  | 1.318*** | 1.836*** | 2.511*** | 2.318*** | 1.282 | 2.313*** | 2.181*** | 1.455*** | 1.697* |
| Mother year 11 education or less | 3.734*** | 1.263* | 1.735*** | 1.612*** | 1.292** | 4.519*** | 1.318*** |  | 1.838*** | 1.801*** | 0.630*** | 2.664*** | 2.640*** | 2.606*** | 1.726*** | 3.070*** |
| Mother zero hours | 2.313*** | 1.157 | 1.562*** | 1.410*** | 1.206* | 2.539*** | 1.836*** | 1.838*** |  | 1.945*** | 1.919*** | 3.922*** | 3.747*** | 3.103*** | 1.416*** | 2.478*** |
| Maternal consistency in lowest quintile | 3.045*** | 1.032 | 1.503*** | 2.006*** | 2.277*** | 1.860*** | 2.511*** | 1.801*** | 1.945*** |  | 1.893*** | 1.910*** | 2.020*** | 1.655*** | 1.635*** | 2.204*** |
| Mother NESB | 0.387*** | 1.142 | 0.671** | 1.112 | 1.041 | 0.292*** | 2.318*** | 0.630*** | 1.919*** | 1.893*** |  | 1.453 | 1.764*** | 1.353** | 1.144 | 3.336*** |
| Four or more siblings | 3.309*** | 1.433 | 1.651** | 0.989 | 1.252 | DNC | 1.282 | 2.664*** | 3.922*** | 1.910*** | 1.453 |  | 0.929 | 3.173*** | 2.289*** | 3.111*** |
| Family in lowest quintile for income | 3.045*** | 1.546** | 1.338** | 1.384** | 1.352** | 4.595*** | 2.313*** | 2.640*** | 3.747*** | 2.020*** | 1.764*** | 0.929 |  | 11.978*** | 1.619*** | 2.332*** |
| Healthcare card | 2.085*** | 1.774*** | 1.497*** | 1.346** | 1.308** | 3.138*** | 2.181*** | 2.606*** | 3.103*** | 1.655*** | 1.353** | 3.173*** | 11.978*** |  | 1.868*** | 2.947*** |
| Most disadvantaged quintile for neighbourhood disadvantage | 2.988*** | 1.233 | 1.533*** | 1.283* | 0.916 | 2.083*** | 1.455*** | 1.726*** | 1.416*** | 1.635*** | 1.144 | 2.289*** | 1.619*** | 1.868*** |  | 1.837*** |
| Child not read to all in last week | 2.116* | 1.843** | 2.040*** | 2.073*** | 1.285 | 1.198 | 1.697* | 3.070*** | 2.478*** | 2.204*** | 3.336*** | 3.111*** | 2.332*** | 2.947*** | 1.837*** |  |

*P < 0.05; **P < 0.01; P < 0.001***; n.s. non-significant, DNC = did not converge

Figure 2 describes the distribution of risks observed within each of the classes, based on assigning children based on maximum posterior probability. Group 1 is the only group which contained children with zero risks. Although some distributions showed similar mid-points (such as child and psychological factors and NESB-and-risk), the shapes of distributions varied.

**Figure 2. Distribution of risks, 6-class LCA.**
