# Supplementary material for: Patterns of Multiple Risk Exposures for Low Receptive Vocabulary Growth 4-8 Years in the Longitudinal Study of Australian Children
Source: PLoS One. 2017 Jan 23;12(1):e0168804. doi: 10.1371/journal.pone.0168804 (PMC5256896; doi:10.1371/journal.pone.0168804)
Supplement: S3 Appendix — (DOCX) [file pone.0168804.s003.docx]

# S3 Appendix: Model fit

**Table 1. Model Fit.**

| **Number classes** | **LR G sq.** | **DF** | **AIC** | **BIC** | **Entropy** |
| --- | --- | --- | --- | --- | --- |
| 1 | 7723.79 | 65519 | 7755.79 | 7860.01 | 1.00 |
| 2 | 5113.29 | 65502 | 5179.29 | 5394.24 | 0.69 |
| 3 | 4851.09 | 65485 | 4951.09 | 5276.78 | 0.72 |
| 4 | 4654.77 | 65468 | 4788.77 | 5225.19 | 0.75 |
| 5 | 4488.54 | 65451 | 4656.54 | 5203.7 | 0.64 |
| 6 | 4343.48 | 65434 | 4545.48 | **5203.37** | 0.63 |
| 7 | 4249.71 | 65417 | 4485.71 | 5254.34 | 0.62 |
| 8 | 4176.16 | 65400 | 4446.16 | 5325.52 | 0.63 |
| 9 | 4113.21 | 65383 | 4417.21 | 5407.3 | 0.68 |
| 10 | 4062.77 | 65366 | 4400.77 | 5501.6 | 0.67 |
| 11 | 4018.73 | 65349 | **4390.73** | 5602.3 | 0.68 |
| 12 | 3988.5 | 65332 | 4394.5 | 5716.8 | 0.69 |
